# Seco-Duocarmycin SA Augments the Impact of Proton Radiation on Human Glioblastoma Cells

**DOI:** 10.3390/ijms27031532

**Published:** 2026-02-04

**Authors:** Ann Morcos, Joab Galvan Bustillos, Yeonkyu Jung, Ryan N. Fuller, Antonella Bertucci, David Caba Molina, Amy Nguyen, Quanqing Zhang, Kristopher E. Boyle, William H. R. Langridge, Marcelo Vazquez, Nathan R. Wall

**Affiliations:** 1 Department of Radiation Medicine, James M. Slater, MD Proton Treatment & Research Center, Loma Linda University Health, Loma Linda, CA 92350, USA; amorcos@llu.edu (A.M.); jgalvanbustillos@llu.edu (J.G.B.); david.jung@agilent.com (Y.J.); antonella.bertucci@cnl.ca (A.B.); 2 Division of Human Anatomy, Department of Pathology and Human Anatomy, Loma Linda University School of Medicine, Loma Linda, CA 92350, USA; 3 Division of Surgical Oncology, Department of Surgery, Riverside University Health System—University of California Riverside, Moreno Valley, CA 92555, USA; dcabamolina@llu.edu; 4 Department of Biological Sciences, California Baptist University, Riverside, CA 92504, USA; ryfuller@calbaptist.edu; 5 Nuclear Response & Analysis, Canadian Nuclear Laboratories, Chalk River, ON K0J 1J0, Canada; 6 Proteomics Core, Institute for Integrative Genome Biology, University of California, Riverside, CA 92521, USA; amy.nguyen010@email.ucr.edu (A.N.); quanqinz@ucr.edu (Q.Z.); 7 School of Pharmacy, Loma Linda University, Loma Linda, CA 92350, USA; kboyle@llu.edu; 8 Division of Biochemistry, Department of Basic Sciences, Loma Linda University School of Medicine, Loma Linda, CA 92350, USA; blangridge@llu.edu; 9 Radiobiology & Health, Canadian Nuclear Laboratories, Chalk River, ON K0J 1J0, Canada

**Keywords:** glioblastoma multiforme, seco-DSA, proton radiation, potency, synergy, cellular mechanisms

## Abstract

Glioblastoma multiforme (GBM) is an aggressive brain tumor with limited treatment options and poor survival outcomes. This study evaluated the anticancer potential of seco-duocarmycin SA (seco-DSA), a potent DNA-alkylating agent, alone and in combination with proton radiation in human GBM cell lines. Human glioblastoma cell lines T98G and LN18 were treated with varying concentrations of seco-DSA, proton radiation doses (2, 4, or 8 Gy), or both. Proton irradiation was delivered with a 250-MeV beam. Clonogenic survival, cell proliferation, and cell cycle distribution were analyzed using colony formation and flow cytometry assays. Proteomic analysis of LN18 cells was performed by LC-MS/MS followed by bioinformatic pathway analysis. Statistical significance was determined using a two-tailed unpaired *t*-test (*p* ≤ 0.05), and Bliss synergy scores were calculated to assess treatment interactions. Combination therapy produced additive and synergistic inhibition of colony formation and enhanced G2/M phase arrest compared with either treatment alone. Apoptosis and necrosis increased modestly but did not fully account for observed cytotoxicity. Proteomic profiling revealed differential expression of proteins involved in DNA repair, apoptosis, and senescence, indicating that seco-DSA broadened radiation-induced stress responses. Seco-DSA potentiates the cytotoxic effects of proton radiation in GBM cells through enhanced clonogenic inhibition and modulation of cell cycle and DNA repair pathways. These findings support seco-DSA as a promising radiosensitizer for further preclinical evaluation.

## 1. Introduction

Glioblastoma multiforme (GBM), also referred to as Grade IV astrocytoma, is one of the deadliest and most aggressive forms of primary brain tumors, accounting for approximately 15% of all primary brain tumors and 60% of astrocytomas [1]. Despite significant research efforts, advancements in effective therapies for GBM have been limited. Standard treatment protocols typically include surgical resection, followed by adjuvant radiotherapy and chemotherapy; yet, survival outcomes remain poor, with a five-year survival rate below 10% [2,3]. Current therapies provide limited benefits due to GBM’s highly invasive nature and its capacity for resistance to chemotherapeutic agents and radiation [4], underscoring the critical need for innovative approaches to enhance treatment efficacy and minimize adverse effects.

Recent progress in radiotherapy has emphasized particle beam therapy, including proton radiation, as an important form of conformal radiation therapy for GBM and other cancers [5]. Proton radiation causes DNA damage and, due to its physical characteristics, delivers a majority of the radiation dose to the tumor location at the desired depth, reducing damage to healthy surrounding tissue [6]. Past research has illustrated that, in comparison with conventional radiation, proton radiation is 10% more effective at eliminating cancer cells when treated with the same dose of radiation [7]. Accelerated protons are advantageous because the maximum energy deposited in unit mass, also known as Bragg’s peak, is delivered at the tumor target site without having an exit route [7]. The width of Bragg’s peak can be manipulated to deliver the maximum radiation dose at the tumor site, minimizing the deposition of radiation dose to healthy tissues surrounding the cancer cells.

In addition to radiotherapy, chemotherapeutic agents such as the alkylating agent temozolomide (TMZ) are commonly employed as radiosensitizers in GBM treatment to augment the effectiveness of the radiation therapy [2]. However, these agents are often limited by systemic toxicity, resistance, and adverse side effects [8]. Therefore, there is a significant need to explore novel approaches for increasing the radiosensitivity of GBM cells. Surgery remains an important component of GBM treatment, offering the most significant impact on survival, yet not all tumors are amenable to resection, and residual tumor cells always persist postoperatively [9]. Safer and more effective therapies are needed to slow disease progression and improve overall survival while minimizing side effects, ultimately improving patient outcomes and quality of life.

This study provides a quantitatively informed evaluation of seco-duocarmycin stable A (seco-DSA) as a proton radiation radiosensitizer in GBM. Seco-DSA, an exceptionally potent member of the duocarmycin family, has shown significant antitumor activity through its sequence-selective alkylation of duplex DNA at AT-rich sites, binding to the DNA minor groove and alkylating adenine residues at the N3 position [10]. Despite potent cytotoxicity at picomolar concentrations, the clinical utility of the duocarmycin family has been constrained by severe hepatotoxicity and myelotoxicity [11]. Recent developments in antibody-drug conjugates (ADCs) possess the potential to address these limitations by employing DSA as a cytotoxic payload, which can potentially improve targeting and minimize systemic toxicity [12].

In this paper we explore the effect of combining seco-DSA with proton radiation in human GBM cell lines T98G and LN18. We hypothesize that by pairing seco-DSA with proton radiation, a more dynamic effect will result, amplifying their collective power and unleashing an enhanced cytotoxicity against human GBM cells. Our experimental results demonstrate that the combination of seco-DSA and radiation leads to both additive and synergistic enhancements in colony inhibition and proliferation arrest, depending on the specific dose and concentration used in both T98G and LN18 cells. Additionally, this combination induces a greater G2/M phase arrest in comparison with radiation or seco-DSA treatment alone. When treated with radiation alone, no significant changes in apoptosis or necrosis were observed, except at the 8 Gy dose in LN18 cells. In all cases, increases in apoptosis and necrosis were insufficient to explain the cytotoxic effects observed. Exploratory proteomic analysis of seco-DSA combined with proton radiation indicated distinct proteomic signatures and engaged broader stress-related pathways, particularly at lower radiation doses, highlighting seco-DSA’s ability to modulate and expand the cellular response beyond what is seen with radiation alone. These findings provide pathway-level context for understanding the mechanism of synergy within these cells.

## 2. Results

### 2.1. Seco-DSA Enhances Radiation-Induced Clonogenic Inhibition in T98G and LN18 Cells

We first aimed to determine the response of human glioblastoma cell lines T98G and LN18 to radiation to assess their relative sensitivity to proton radiation as a stand-alone treatment. We used two models to quantify each cell line’s response to radiation: the single-target/single-hit model (STM) and the linear-quadratic model (LQM).

**Figure 1:**
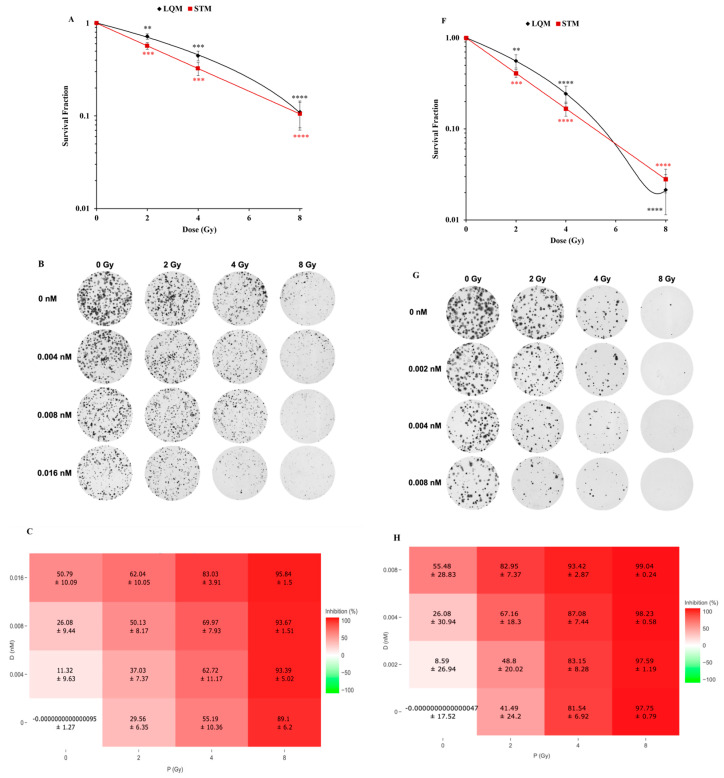

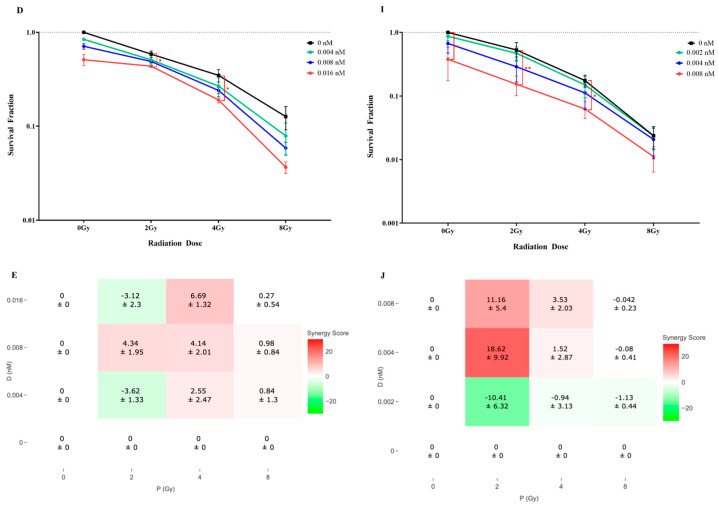
Proton radiation reduces glioblastoma cell survival and synergizes with seco-DSA to enhance cytotoxicity. T98G and LN18 cells were treated with seco-DSA 8 h prior to proton irradiation and assessed by colony formation assay two weeks post-irradiation. (**A**) Radiation dose-response curve for T98G cells. Survival fraction was plotted using both the linear-quadratic (LQM) and single-target/single-hit model (STM). Black diamonds and red squares represent survival fractions associated with LQM and STM modeling approaches, respectively, with solid lines indicating the corresponding model fits. (Table 1) Fitted parameters: α = 0.128 Gy^−1^, β = 0.019 Gy^−2^, α/β = 6.9 Gy; D_50_ = 2.5 Gy. (**B**) Representative images of stained T98G colonies following treatment. (**C**) Percent inhibition of T98G cell proliferation following proton radiation (Gy), alone or in combination with seco-DSA (nM), relative to untreated control. (**D**) Survival fraction of T98G cells following proton radiation, alone or in combination with seco-DSA, relative to untreated control. (**E**) Bliss synergy score heatmap showing synergistic interactions between seco-DSA (nM) and proton radiation (Gy) in T98G cells. (**F**) Radiation dose-response curve for LN18 cells fitted with LQM and STM models. (Table 2) Parameters: α = 0.23 Gy-1, β = 0.032 Gy-2, α/β = 7.2 Gy; D_50_ = 1.6 Gy. (**G**) Representative images of stained LN18 colonies following treatment. (**H**) Percent inhibition of LN18 cell proliferation following proton radiation (Gy), with or without seco-DSA (nM), relative to untreated control. (**I**) Survival fraction of LN18 cells following proton radiation, alone or in combination with seco-DSA, relative to untreated control. (**J**) Bliss synergy score heatmap showing synergistic interactions between seco-DSA (nM) and proton radiation (Gy) in LN18 cells. Statistical significance was assessed using the following criteria: * *p* < 0.05, ** *p* < 0.01, *** *p* < 0.001, and **** *p* < 0.0001 compared to the control group.

STM is a simple model based on the assumption that a cell dies when a single lethal event is sufficient to inactivate clonogenic survival. In this model, D_0_ is defined as the dose (in Gy) that delivers, on average, one lethal event per target, reducing the surviving fraction from 100% to 37% [13]. This model is useful for estimating D_50_, the dose required to reduce survival to 50% in a colony formation assay. In our experiments, the D_50_ for T98G was measured to be 2.5 Gy (Figure 1A and Table 1), while for LN18, it was measured to be 1.6 Gy (Figure 1F and Table 2). This suggests that LN18 is more sensitive to proton radiation than T98G.

LQM is considered the gold standard model in radiotherapy because it provides a robust quantification of both single-event (α, linear) and multi-event (β, quadratic) cell killing. The shape of the survival curve is determined by the α/β ratio [13], where α represents the linear, non-repairable damage (typically double-strand DNA breaks) and is expressed in units of Gy^−1^. β represents the quadratic, repairable damage, often associated with interactions between single-strand breaks, and is expressed in units of Gy^−2^ [14]. At low doses, most cell killing results from α-type (single-hit, non-repairable) damage, whereas at higher doses, β-type (multi-hit, repairable) damage becomes more dominant, increasing proportionally to the square of the dose [15]. The α/β ratio represents the dose in Gy at which linear and quadratic contributions to cell killing are equal [16]. A high α/β ratio indicates that the cell line is more sensitive to total dose, with the linear α component dominating; this means that the biological effect is similar whether the dose is delivered all at once or in fractions. In contrast, a low α/β ratio implies greater sensitivity to fractionated doses, with a more prominent quadratic (β) component and greater capacity for sublethal damage repair, typically seen in normal tissues or late-responding cells [17].

We calculated an α/β ratio of 6.9 Gy for T98G and 7.2 Gy for LN18 (Figure 1A,F, Table 1 and Table 2), which is considered relatively high for both cell lines. This data suggests that whether the dose is delivered in a single exposure or fractionated, the biological effect remains largely the same, as T98G and LN18 cells exhibit limited sublethal DNA damage repair. This behavior is characteristic of many cancer cells.

The combined effects of seco-DSA and proton radiation on T98G and LN18 cells in a colony formation assay were assessed by treating cells with increasing concentrations of seco-DSA and radiation, based on previously determined IC_50_ values [18]. Figure 1B,G shows representative images of crystal violet–stained colonies across treatments, illustrating the effects of radiation alone, seco-DSA alone, and their combination on colony inhibition. The corresponding inhibition percentages and survival fractions are quantified in Figure 1C,D,H,I.

In both T98G and LN18 cell lines, combining seco-DSA with radiation resulted in increased colony inhibition across all radiation doses. This effect was particularly significant when 0.016 nM seco-DSA (for T98G) and 0.008 nM seco-DSA (for LN18) were combined with 2 Gy and 4 Gy of radiation (Figure 1D,I). The impact of combination treatment was less pronounced at 8 Gy in both cell lines, likely because radiation alone already caused substantial cytotoxicity, limiting further measurable inhibition (Figure 1C,H).

To assess whether combining seco-DSA with proton radiation resulted in a synergistic effect, Bliss synergy scores were calculated using SynergyFinder Plus [19]. While radiation and seco-DSA both induce DNA damage through distinct mechanisms, Bliss independence is used here as a descriptive probabilistic framework and does not resolve whether downstream DNA damage responses interact mechanistically. A Bliss score greater than 10 indicates synergy, while a score between −10 and 10 suggests an additive effect. In T98G cells, Bliss synergy analysis (Figure 1E) revealed that the combination treatments were additive across all doses. The strongest additive effects were observed at 4 Gy across all three seco-DSA concentrations and at 2 Gy when combined with 0.008 nM seco-DSA. In LN18 cells, synergy was observed when 0.004 nM and 0.008 nM seco-DSA were combined with 2 Gy, while all other combinations produced additive effects.

### 2.2. Combination Treatment with Seco-DSA and Proton Radiation Reduces Proliferation and Induces Synergy in Glioblastoma Cells

**Figure 2:**
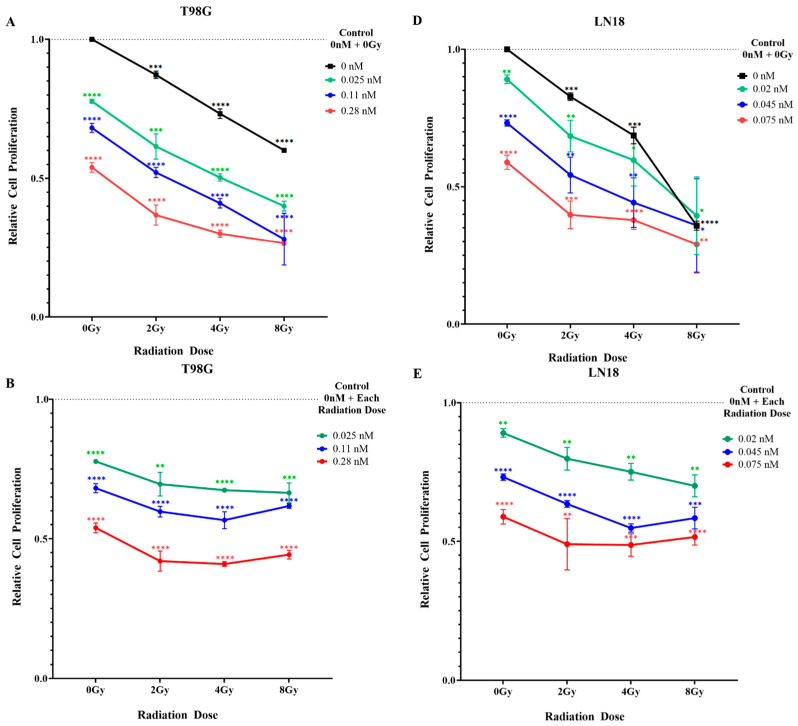

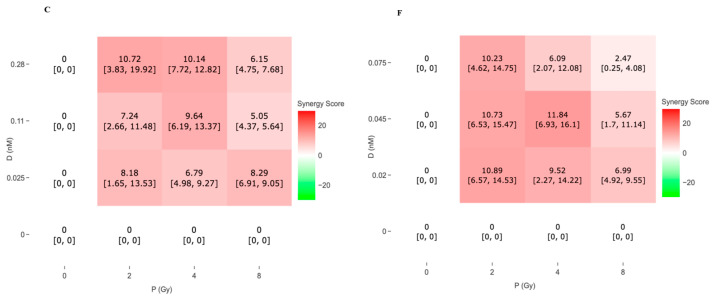
Combined Treatment with Seco-DSA and Proton Radiation Synergistically Reduces Proliferation of Glioblastoma Cells. T98G and LN18 cells were treated with increasing doses of proton radiation in combination with Seco-DSA. Seco-DSA was administered 8 h prior to irradiation and remained present throughout the experiment. Seventy-two hours after radiation, cell proliferation was assessed. (**A**) Relative proliferation of T98G cells compared to untreated controls. (**B**) Relative proliferation of T98G cells following combination treatment with Seco-DSA, normalized to radiation-only treatment at each corresponding dose. (**C**) Bliss synergy score heatmap for T98G cells treated with proton radiation (Gy) and Seco-DSA (nM). Positive scores indicate synergy, with the highest observed at 0.28 nM Seco-DSA combined with 2 Gy radiation (score: 10.72). (**D**) Relative proliferation of LN18 cells compared to untreated controls. (**E**) Relative proliferation of LN18 cells following combination treatment with Seco-DSA, normalized to radiation-only treatment at each corresponding dose. (**F**) Bliss synergy score heatmap for LN18 cells. Positive synergy scores were observed across most conditions, with maximal synergy at 0.045 nM Seco-DSA and 2 Gy radiation (score: 11.84). Data were normalized to control and are expressed as the average ± SE from three independent experiments. Statistical significance was assessed using the following criteria: * *p* < 0.05, ** *p* < 0.01, *** *p* < 0.001, and **** *p* < 0.0001 compared to the control group.

The current standard therapeutic radiation treatment for glioblastoma consists of daily 2 Gy fractions administered to the patient over a six-week period, delivering a total dose of 60 Gy [20]. To model this clinically relevant exposure, we examined the effects of a single 2 Gy proton dose on T98G and LN18 glioblastoma cells to assess their immediate molecular and cellular responses. These insights may inform strategies to enhance the therapeutic efficacy of the daily dose limit used in standard radiotherapy. Four and 8 Gy doses were also included to model the effects of radiation-mediated cytotoxicity (Figure 2A,D).

T98G cells exhibited a dose-dependent reduction in proliferation following proton radiation. Compared to untreated controls, proliferation decreased to 0.87 at 2 Gy, 0.73 at 4 Gy, and 0.60 at 8 Gy (Figure 2A). Similarly, seco-DSA treatment induced a concentration-dependent decrease in proliferation, with values of 0.78 at 0.025 nM, 0.68 at 0.11 nM, and 0.54 at 0.28 nM.

At the lowest tested concentration of seco-DSA (0.025 nM), proliferation was reduced to 0.78, similar to the level observed with 4 Gy proton radiation (0.73). When this concentration of seco-DSA was combined with 2 Gy radiation, proliferation was further reduced to 0.61, closely approximating the effect of 8 Gy radiation (0.60). At the IC_50_ concentration of seco-DSA (0.28 nM), proliferation decreased to 0.54. When combined with 2 Gy, 4 Gy, or 8 Gy proton radiation, proliferation further declined to 0.30, 0.30, and 0.27, respectively (Figure 2A,B). These results suggest that the combination of 2 Gy proton radiation and seco-DSA at its IC_50_ concentration is sufficient to achieve near-maximal suppression of proliferation, with higher radiation doses yielding marginal additional effects.

To determine whether the combined treatment of seco-DSA and proton radiation on T98G cells produced a synergistic effect, Bliss synergy analysis was performed. Positive synergy scores were observed across all tested combinations, indicating an additive interaction between seco-DSA and proton radiation in T98G cells at the 0.025 nM and 0.11 nM concentrations when combined with proton radiation (Figure 2C). Synergy was observed at 0.28 nM seco-DSA combined with 2 Gy proton radiation (score: 10.72 (3.83, 19.92)) and with 4 Gy (10.14 (7.72, 12.82)). These results indicate that seco-DSA enhances the antiproliferative effects of proton radiation through a synergistic mechanism when 0.28 nM (IC_50_) is combined with low radiation doses.

LN18, a more radiosensitive glioblastoma cell line compared to T98G, also exhibited a dose-dependent reduction in proliferation following proton radiation. Compared to untreated controls, proliferation decreased to 0.83 at 2 Gy, 0.69 at 4 Gy, and 0.36 at 8 Gy (Figure 2D,E). Similarly, seco-DSA treatment alone induced a concentration-dependent decrease in proliferation, with values of 0.89 at 0.02 nM, 0.78 at 0.045 nM, and 0.59 at 0.075 nM. When the IC_50_ concentration of seco-DSA (0.075 nM) was combined with 2 Gy proton radiation, proliferation was reduced to 0.39, comparable to that observed with 8 Gy radiation alone. At 8 Gy, the addition of seco-DSA had minimal impact, likely because radiation alone had already induced near-maximal cytotoxicity, leaving limited room for further suppression.

To assess synergy in LN18 cells, Bliss synergy analysis was performed across the range of drug and radiation dose combinations detailed above (Figure 2F). Positive synergy scores were observed throughout the matrix, confirming both additive and synergistic interaction between seco-DSA and proton radiation in this radiosensitive glioblastoma line. The strongest synergy was observed at the 2 Gy combination treatment, with all three concentrations + 2 Gy resulting in scores greater than 10. In contrast, synergy scores declined at higher radiation doses, particularly at 8 Gy, where a ceiling effect of radiation-induced cytotoxicity likely limited the additive contribution of seco-DSA. These findings suggest that combining seco-DSA with low proton radiation may yield optimal therapeutic synergy in radiosensitive glioblastoma cells.

### 2.3. Combined Treatment of Seco-DSA with Proton Radiation-Induced G1-Phase Depletion and G2/M-Phase Arrest in T98G and LN18 Cells Compared to Either Treatment Alone

To determine whether the observed decrease in live cell count resulted from cell cycle phase arrest and to identify which phases were affected, T98G and LN18 cells were treated with graded doses of radiation alone, the IC_50_ concentration of each cell line’s seco-DSA, or a combination of IC_50_ seco-DSA with each radiation dose.

**Figure 3:**
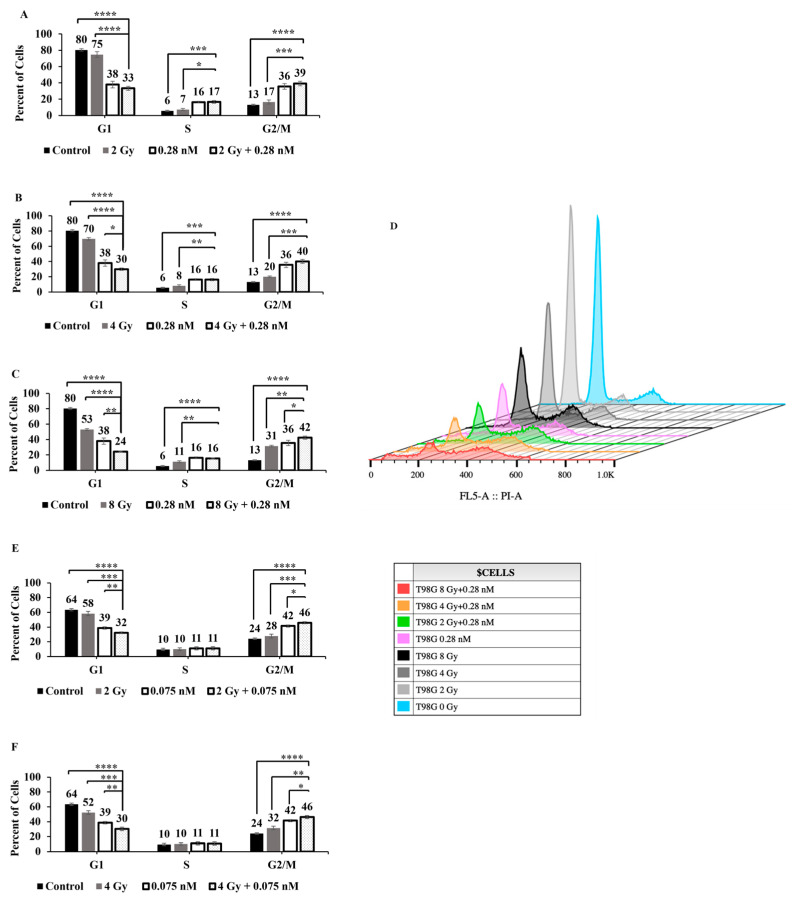

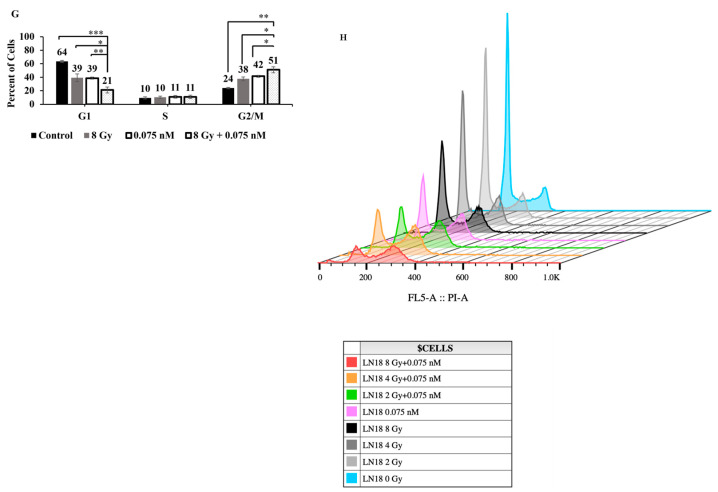
Cell cycle progression changes induced by proton radiation alone, seco-DSA alone, and seco-DSA combined with a gradient dose of proton radiation. Cells were irradiated 8 h after adding seco-DSA, and cell cycle phases were analyzed by flow cytometry using propidium iodide at 72 h post-treatment. In panels (**D**,**H**), the *x*-axis (FL5-A:: PI-A) represents propidium iodide fluorescence intensity corresponding to cellular DNA content, and the y-axis represents relative cell count. In the bar graphs, the *y*-axis represents the percentage of cells, and the *x*-axis represents the different phases of the cell cycle. Color coding is used to identify the different treatment groups. The black bars represent the untreated control cells treated with 0.5% DMSO, the gray bars represent the radiation-only groups, the white bars represent the seco-DSA alone group at the respective IC50 concentration for each cell line, and the dotted bars represent the proton radiation plus seco-DSA groups. The displayed analysis focuses on the comparison of the radiation plus seco-DSA groups to the other treatment groups. The histograms show the raw cell cycle data from FlowJo, with peaks in the G1-, S-, and G2/M-phases. (**A**) T98G 2 Gy plus seco-DSA. (**B**) T98G 4 Gy plus seco-DSA. (**C**) T98G 8 Gy plus seco-DSA. (**D**) T98G cells raw FlowJo data, color coding indicates each treatment group. (**E**) LN18 2 Gy plus seco-DSA. (**F**) LN18 4 Gy plus seco-DSA. (**G**) LN18 8 Gy plus seco-DSA. (**H**) LN18 cells raw FlowJo data, color coding indicates each treatment group. Each bar represents the mean percentage of cells. The data are expressed as the average ±SD from three independent experiments in triplicate. Statistical significance was assessed using the following criteria: * *p* < 0.05, ** *p* < 0.01, *** *p* < 0.001, and **** *p* < 0.0001.

As shown in Figure 3, radiation alone induced a dose-dependent decrease in the G1-phase population in both cell lines. In T98G cells, G1-phase cells decreased from 80% in control to 75% at 2 Gy, 70% at 4 Gy, and 53% at 8 Gy (Figure 3A–D). Similarly, in LN18 cells, G1-phase cells declined from 64% in control to 58% at 2 Gy, 52% at 4 Gy, and 39% at 8 Gy (Figure 3E–H). Both cell lines also exhibited a dose-dependent increase in the G2/M population. In LN18, G2/M-phase cells increased from 24 percent in control to 28, 32, and 38 percent in response to 2, 4, and 8 Gy, respectively. In T98G, G2/M increased from 13 percent in control to 17, 20, and 31 percent, respectively. These results suggest that higher radiation doses lead to a significant depletion of G1-phase cells and accumulation in the G2/M-phase, indicating G2/M arrest.

Treatment with seco-DSA alone caused approximately 50% reduction in G1-phase cells in T98G and approximately 40% reduction in LN18, compared to their respective controls. G2/M-phase arrest was also evident, with the population increasing from 13% to 36% in T98G and from 24% to 42% in LN18. Notably, seco-DSA induced a substantial increase in the S-phase population in T98G, from 6% to 16%, whereas LN18 showed minimal change in S-phase under the same treatment. This may be significant, as the S phase in the cell cycle is known to be resistant to radiation therapy [21].

When seco-DSA was combined with radiation, both cell lines exhibited a further reduction in the G1-phase population, significantly beyond levels seen with either treatment alone. This effect was most pronounced at the 8 Gy dose, where G1-phase dropped to 24% in T98G and 21% in LN18. These data indicate that the combination of radiation and drug more effectively depletes G1-phase cells than either stimulus alone.

In LN18 cells, the S-phase population remained essentially unchanged when seco-DSA was combined with proton radiation, consistent with earlier observations. In T98G, the S-phase population remained elevated under combination treatment, similar to levels seen with seco-DSA alone. Although this elevation was significantly higher than with radiation alone, it did not increase further with higher radiation doses, suggesting that the S-phase effect is primarily drug driven.

A notable G2/M-phase arrest was observed in both cell lines under the combination treatment, especially in LN18 cells, where the combination of any radiation dose with seco-DSA led to significantly greater G2/M accumulation than either treatment alone. In T98G cells, combination treatment induced a statistically significant increase in G2/M-phase compared to all doses of radiation alone. A significant difference relative to seco-DSA alone was only seen in the 8 Gy plus seco-DSA group.

In summary, the combination of seco-DSA with proton radiation resulted in pronounced G1 depletion and G2/M-phase arrest in both T98G and LN18 cells. LN18 cells displayed a stronger G2/M arrest response, consistent with their greater sensitivity to both radiation and seco-DSA, while T98G cells showed a significant, though comparatively smaller, G2/M-phase arrest.

### 2.4. The Significant Decrease in Live Cell Count Is Not Fully Attributable to Apoptosis and Necrosis

To clarify the extent to which the decrease in live cell counts resulted from apoptosis and/or necrosis, LN18 and T98G cells were treated with 2, 4, and 8 Gy doses of proton radiation, with and without their respective IC_50_ of seco-DSA (0.075 nM for LN18 and 0.28 nM for T98G). Cells were treated with seco-DSA, radiated eight hours after treatment, and left to incubate for 72 h, after which they were collected and stained with Annexin-V and 7AAD. The IC_50_ and incubation time were determined based on our previous publication, which investigated in detail the effects of seco-DSA in both cell lines [18].

**Figure 4:**
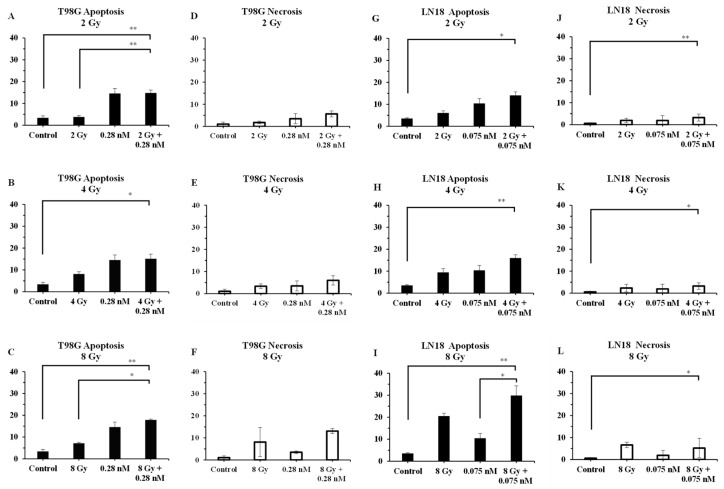
The significant decrease in live cell count is not fully attributable to apoptosis and necrosis. Flow cytometric analysis of apoptosis and necrosis in T98G and LN18 cells treated with increasing doses of proton radiation (2, 4, or 8 Gy), with or without seco-DSA (0.28 nM for T98G and 0.075 nM for LN18). After 72 h, cells were stained with Annexin V and 7-AAD and analyzed by flow cytometry. The y-axis represents the percentage of necrotic (AV−/7AAD+) or apoptosis (AV+/7AAD− and AV+/7AAD+) cells. The x-axis represents the analyzed groups: control, radiation alone, seco-DSA alone, and radiation plus seco-DSA. The displayed analysis represents a comparison between the radiation plus seco-DSA group and all other groups. The controls were untreated and received vehicle only (0.5% DMSO), and the other groups were treated as indicated by the labels on the *x*-axis. (**A**) T98G cells apoptosis in 2 Gy treatment groups. (**B**) T98G cells apoptosis in 4 Gy treatment groups. (**C**) T98G cells apoptosis in 8 Gy treatment groups. (**D**) T98G cells necrosis 2 Gy. (**E**) T98G cells necrosis 4 Gy. (**F**) T98G cells necrosis 8 Gy. (**G**) LN18 apoptosis 2 Gy treatment groups. (**H**) LN18 apoptosis 4 Gy treatment groups. (**I**) LN18 apoptosis 8 Gy treatment groups. (**J**) LN18 necrosis 2 Gy treatment groups. (**K**) LN18 necrosis 4 Gy treatment groups. (**L**) LN18 necrosis 8 Gy treatment groups. Each bar represents the mean percentage of cells, and data are expressed as the average ± SD from three independent experiments performed in triplicate. Statistical significance is displayed using the following criteria: * *p* < 0.05, ** *p* < 0.01.

In T98G cells, apoptosis increased with radiation in a dose-dependent manner, and the addition of seco-DSA further enhanced this effect (Figure 4A–C). Apoptotic cell populations statistically increased from 4% with 2 Gy alone to 15% with 2 Gy + seco-DSA, and from 7% with 8 Gy alone to 18% with 8 Gy + seco-DSA. These differences were statistically significant, but overall apoptotic levels remained modest. seco-DSA alone induced levels of apoptosis that were comparable to those seen in the combination groups. This suggests that the addition of radiation does not markedly enhance apoptosis beyond what is induced by the IC_50_ of seco-DSA alone.

Necrosis also increased slightly for T98G in the combination groups compared to radiation alone, but at 2 Gy and 4 Gy, necrotic cell population remained below 6.5% (Figure 4E,G). Necrosis increased significantly at the 8 Gy dose when combined with seco-DSA, reaching 13%. This was substantially higher than necrosis with 8 Gy alone at 8% or seco-DSA alone at 3.5% (Figure 4G). Although necrosis in T98G cells increased significantly at 8 Gy with seco-DSA, reaching 13%, this still represents a relatively low percentage of total cells, especially given the use of both the IC_50_ concentration of seco-DSA and a high radiation dose.

In LN18 cells, apoptosis showed a dose-dependent response to proton radiation, increasing from 6% in the 2 Gy group to 9% in the 4 Gy group and 21% in the 8 Gy group. This effect was enhanced with the addition of seco-DSA, reaching 14% in the 2 Gy plus seco-DSA group and 16% in the 4 Gy plus seco-DSA group. However, this increase was statistically significant only in the 8 Gy combined treatment versus the seco-DSA alone groups (30% vs. 10%). Treatment with radiation alone and seco-DSA alone resulted in a lower percentage of apoptosis compared to the combined treatment at all radiation doses, suggesting an enhancing effect of seco-DSA (Figure 4H–J).

Necrosis showed a modest dose-dependent increase in LN18 cells with radiation alone, going from 2% in the 2 Gy group to 7% in the 8 Gy group. Radiation alone and combined treatment showed similar necrosis percentages (2% vs. 3% for 2 Gy and 4 Gy), suggesting a minimal enhancing effect of seco-DSA. In the 8 Gy group, furthermore, a reduction in necrosis was observed with the addition of seco-DSA, going from 7% with radiation alone to 5% with the combined treatment. No statistical significance in necrosis was found between any of the treatment groups, and the overall necrosis percentage remained below 10% for all comparison groups (Figure 4J–L).

This result suggests that, in both cell lines, necrosis and apoptosis account for only a portion of the cytotoxic response. The low percentage of apoptotic and necrotic cell death observed, despite significant reductions in viability in earlier survival assays, implies that other mechanisms of cell death may be contributing to the overall treatment effect.

### 2.5. Differential Proteomic Signatures in Response to Radiation and Seco-DSA in GBM

**Figure 5:**
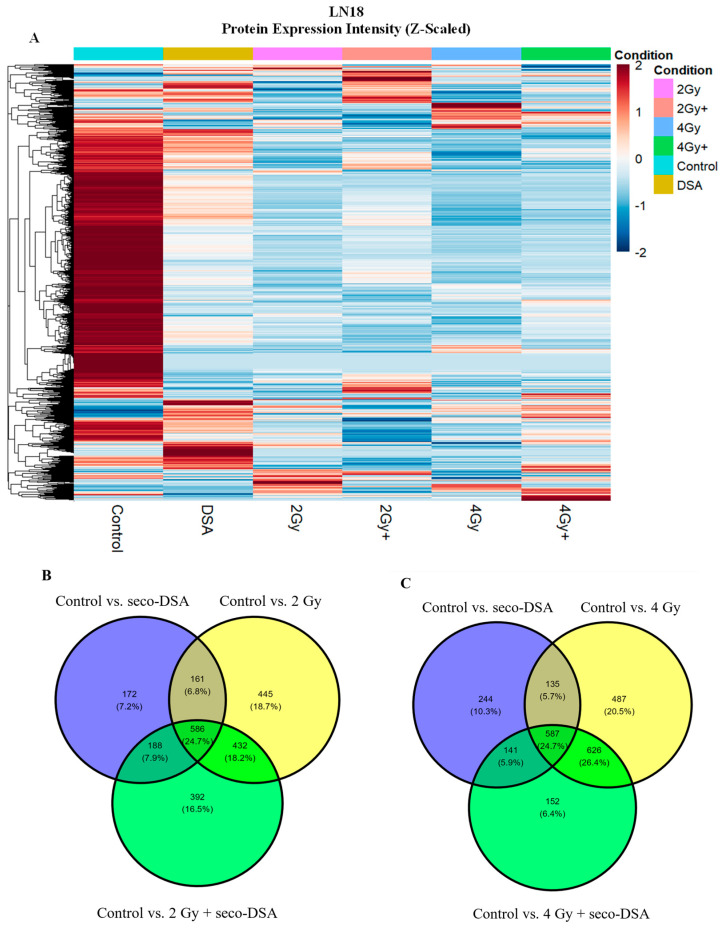

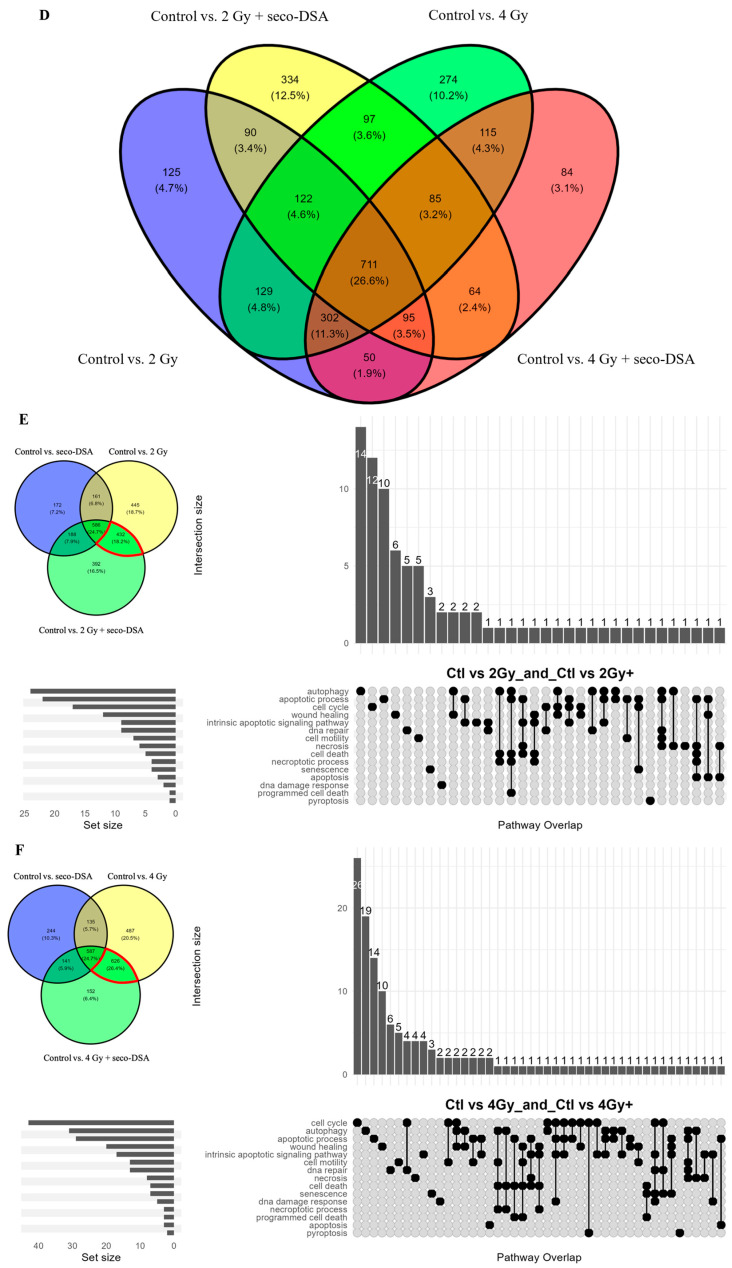

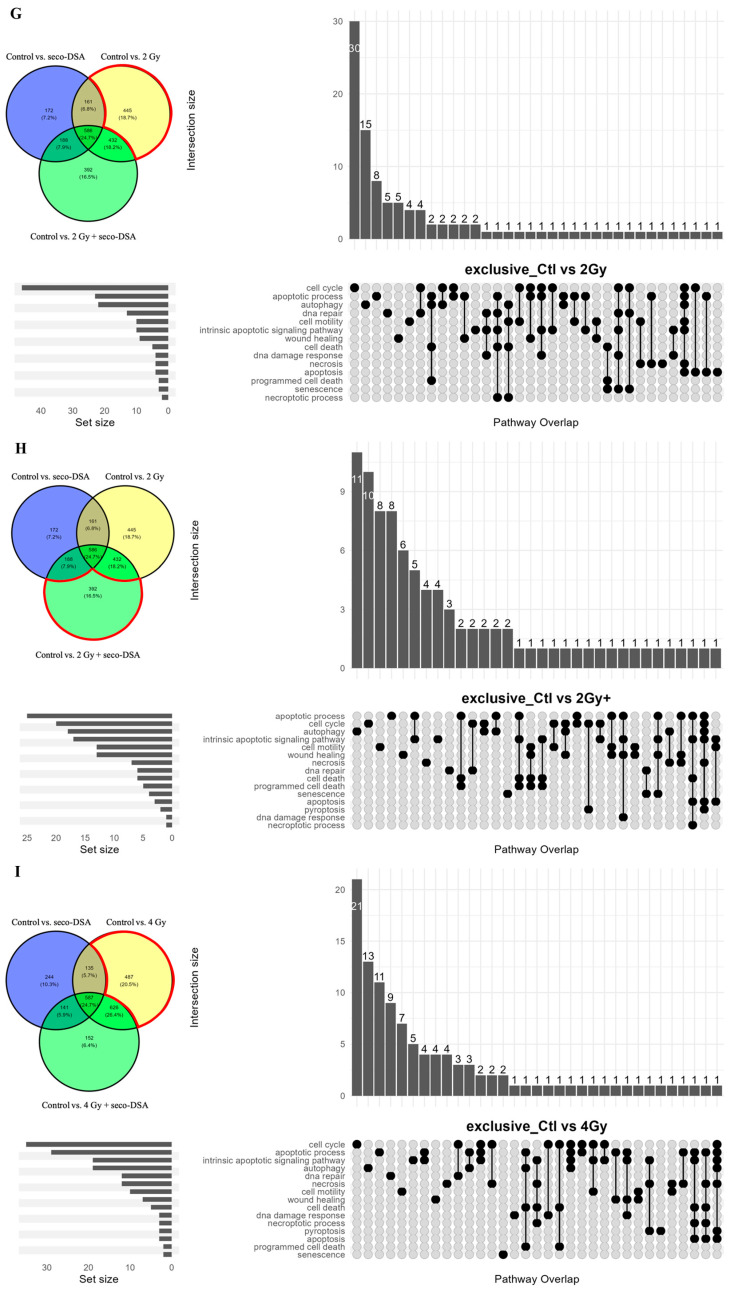

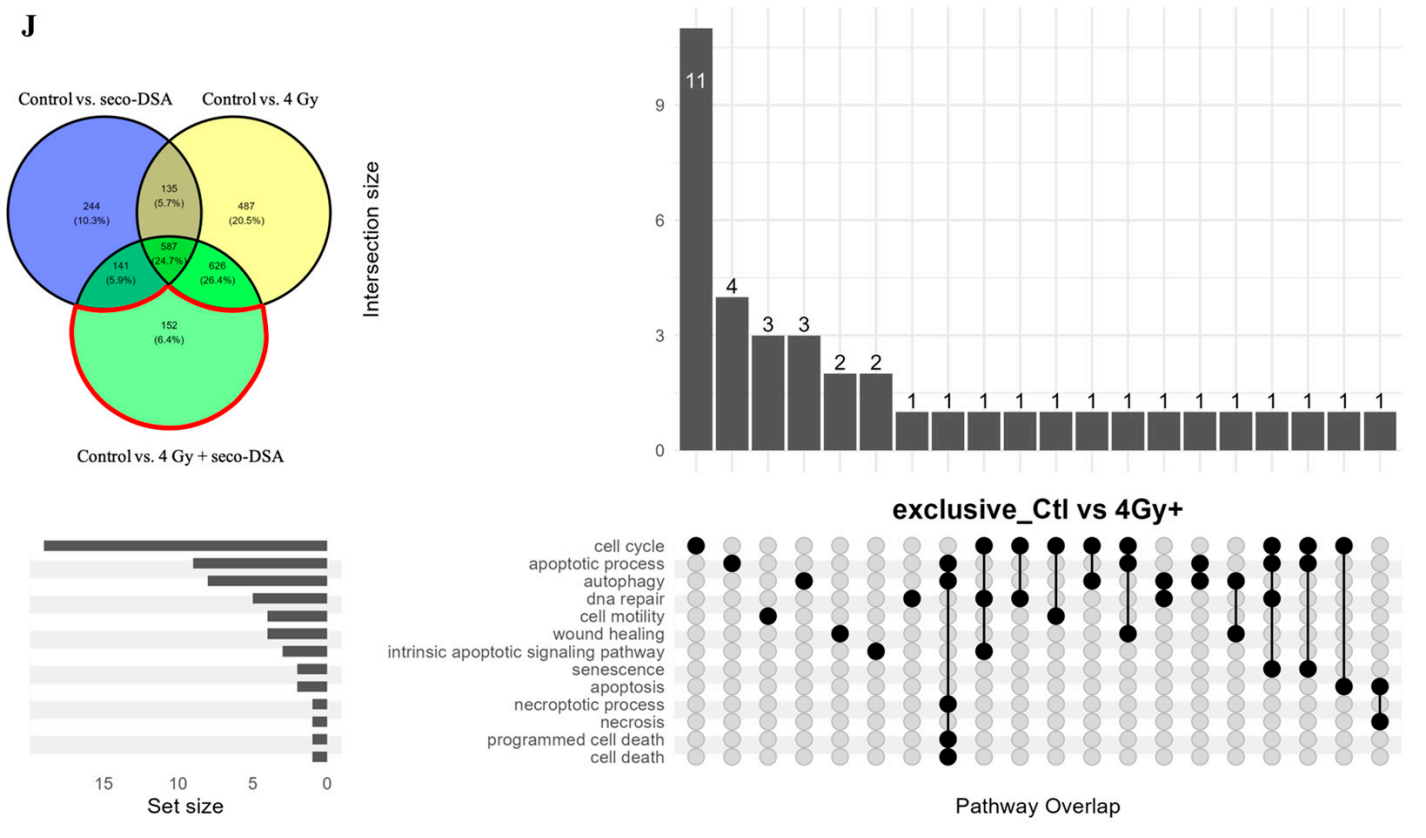
Differential Proteomic Signatures in Response to Radiation and seco-DSA in LN18 Cells. (**A**) Heatmap representing Z-scaled protein expression intensity across different experimental conditions: Untreated control (vehicle only; turquoise), seco-DSA (yellow), 2 Gy (pink), 2 Gy + 0.1 nM seco-DSA (salmon), 4 Gy (blue), 4 Gy + 0.1 nM seco-DSA (green). Condition identity is indicated by the color bar above the heatmap and corresponding labels below. Z-scores represent relative protein expression across conditions, where positive values (red) indicate higher expression and negative values (blue) indicate lower expression relative to the mean expression of each protein. The rows represent the individual protein groups, and the clustering shows the hierarchical relationship between proteins based on similarity in expression patterns across conditions. (**B**,**C**) Numbers inside each section show counts of proteins, and the percentages represent the proportion of total proteins in the comparison. The center overlap (where all three circles intersect) represents proteins common to all three conditions. (**B**) Venn diagram of control vs. seco-DSA (purple), 2 Gy (yellow), and 2 Gy + 0.1 nM seco-DSA (green). (**C**) Venn diagram of control vs. seco-DSA (purple), 4 Gy (yellow), and 4 Gy + 0.1 nM seco-DSA (green). (**D**) Venn diagram showing the overlap of differentially expressed proteins across four treatment conditions compared to control: 2 Gy (purple), 4 Gy (green), 2 Gy + seco-DSA (yellow), and 4 Gy + seco-DSA (pink). (**E**–**J**) UpSet plots showing pathway associations for differentially expressed proteins across treatment conditions involving radiation normalized to the untreated group. The horizontal bar graph on the left represents set size (number of proteins associated with each individual pathway). The vertical bars represent intersection size, indicating the number of proteins shared across pathway combinations, with the *x*-axis corresponding to the intersection categories defined by the dot-matrix columns below. The matrix of connected black dots highlights pathway co-occurrence within shared differentially expressed protein subsets. (**E**) Overlap between control vs. 2 Gy and control vs. 2 Gy + seco-DSA. (**F**) Overlap between control vs. 4 Gy and control vs. 4 Gy + seco-DSA. (**G**) Exclusive differentially expressed proteins in 2 Gy relative to the control. (**H**) Exclusive differentially expressed proteins in 2 Gy + seco-DSA relative to control. (**I**) Exclusive differentially expressed proteins in 4 Gy relative to control. (**J**) Exclusive differentially expressed proteins in 4 Gy + seco-DSA relative to control. (n = 1).

Given the limited contribution of apoptosis and necrosis to overall cytotoxicity, proteomic profiling was used to contextualize the molecular pathways associated with non-lethal stress, checkpoint engagement, and impaired recovery. This proteomic analysis is exploratory and hypothesis-generating in nature, based on a single biological replicate, and is intended to provide associative pathway-level context rather than definitive mechanistic conclusions. Preliminary proteomic analysis was performed on LN18 cells, which were selected over T98G due to their greater sensitivity to seco-DSA and radiation [13]. LN18 cells were treated with seco-DSA 8 h before proton irradiation, then protein was isolated 48 h post-treatment. The primary goal was to identify which cellular pathways were associated across the treatment groups, based on differentially expressed protein clusters associated with pathways of interest, relative to untreated control. First, we aimed to take a broad perspective to assess whether there were major shifts in protein upregulation or downregulation in the treatment groups compared to the control. As shown in Figure 5A, the heatmap based on peak intensity reveals that the control group exhibits large clusters of highly expressed proteins (in red) that are downregulated (in blue) in the treated groups. The heatmap also shows that radiation alone (2 Gy and 4 Gy) generally results in reduced protein expression relative to the control. In contrast, seco-DSA treatment leads to widespread protein upregulation compared to the other groups. Notably, the combination treatments (2 Gy + 0.1 nM seco-DSA and 4 Gy + 0.1 nM seco-DSA) display distinct expression profiles, suggesting they may trigger different or additional pathways compared to either seco-DSA or radiation alone treatments.

We next identified and quantified proteins that were either up- or downregulated in each treatment group, relative to untreated controls, and then these proteins were displayed using Venn diagrams to identify overlaps and singularities in differential protein expression (Figure 5B,C). In the 2 Gy comparison (Figure 5B), 172 proteins were uniquely differentially expressed in the seco-DSA group, while in the 4 Gy comparison (Figure 5C), 244 proteins were uniquely expressed in the seco-DSA group. Similarly, 445 proteins were exclusive to the 2 Gy treatment, and 487 proteins were unique to the 4 Gy treatment. The combination treatments showed 392 unique proteins in the 2 Gy + seco-DSA group and 152 unique proteins in the 4 Gy + seco-DSA group.

We also observed considerable overlap in protein expression profiles within each treatment context. In the 2 Gy Venn diagram (Figure 5B), differential expression of 432 proteins was shared between 2 Gy and 2 Gy + seco-DSA, while 188 proteins shared between seco-DSA and 2 Gy + seco-DSA. Importantly, 586 proteins were shared differential expression across all three treatments (seco-DSA, 2 Gy and 2 Gy + seco-DSA). In contrast, only 172, 445, and 392 proteins were uniquely expressed by seco-DSA, radiation, and the combination of the two, respectively. Thus, a total of 1009 proteins were expressed uniquely, while 1367 were expressed as overlapping treatment arms. This suggests a substantial core of proteins that respond consistently across treatments involving 2 Gy radiation and/or seco-DSA, potentially reflecting common pathways related to DNA damage or stress signaling.

In the 4 Gy Venn diagram (Figure 5C), a similar pattern was observed. A total of 626 proteins were shared between 4 Gy and 4 Gy + seco-DSA, while 141 proteins were shared between seco-DSA and 4 Gy + seco-DSA. Again, 587 proteins were commonly expressed across all three treatments (seco-DSA, 4 Gy, and 4 Gy + seco-DSA). A total of 883 proteins were uniquely expressed, and 1489 were expressed as overlapping treatment arms. This consistent overlap again points to a robust set of shared proteomic responses that may be central to the cell’s reaction to higher radiation doses, either alone or in combination with seco-DSA. These two Venn diagrams based comparison highlight the extent of overlap and exclusivity in differential protein expression across treatment groups, providing a foundational view of the differential proteomic responses.

We compared the number of differentially expressed proteins across the four treatment groups: 2 Gy, 4 Gy, 2 Gy + seco-DSA, and 4 Gy + seco-DSA, to evaluate both shared and treatment-specific responses (Figure 5D). The largest overlap was observed in the set of 711 proteins shared among all four groups (26.6% of the total), reflecting a robust core induced response that is conserved in all four treatments. Interestingly, the 4 Gy group showed more than double the number of unique proteins compared to 2 Gy (274 vs. 125), suggesting that higher radiation doses generate a broader and more distinct stress response. Despite both being radiation-only treatments, 2 Gy and 4 Gy shared only 129 differentially expressed proteins, reinforcing that dose increase significantly alters protein expression. Among the combination treatments, 2 Gy + seco-DSA induced a much larger set of unique proteins compared to 4 Gy + seco-DSA (334 vs. 84). This likely reflects the fact that seco-DSA exerts a more expansive modulatory effect at lower radiation doses, focusing on core damage and checkpoint mechanisms rather than activating broader signaling programs. Notably, the two seco-DSA combination groups shared 64 proteins, suggesting some consistency in how seco-DSA modifies the radiation response, regardless of dose. These findings led us to further investigate pathway-level analysis of interest to interpret the functional significance of these shared and unique protein signatures as seen in the upset plots (Figure 5E–J).

We examined the association of differentially expressed proteins shared between radiation alone and radiation + seco-DSA treatment groups with select pathways of interest, analyzing a total of 432 proteins for the 2 Gy groups and 626 proteins for the 4 Gy groups (Figure 5E,F). Both upset plots reveal a shared radiation-induced stress and cell death signature across the treatment conditions. In the overlap between 2 Gy and 2 Gy + seco-DSA (Figure 5E), the most prominent pathway, based on the number of shared differentially expressed proteins, was autophagy with 14 proteins, followed by cell cycle (12), apoptotic process (10), wound healing (6), DNA repair and cell motility (5), senescence (3), and DNA damage response (2). In the overlap between 4 Gy and 4 Gy + seco-DSA (Figure 5F), a broader and more intense cellular response was observed, with a larger number of shared differentially expressed proteins. The highest number of exclusive proteins was associated with cell cycle (26), autophagy (19), apoptotic process (14), wound healing (10), DNA repair (6), cell motility (5), necrosis and intrinsic apoptotic signaling (4), senescence (3), DNA damage response (2), and apoptosis (2). DNA repair and cell cycle shared 4 pathways. All other pathways had 1–2 overlapping differentially expressed proteins. No proteins associated with ferroptosis were detected in either comparison. These findings suggest that 4 Gy and 4 Gy + seco-DSA are associated with a more diverse and extensive set of enriched pathways than the 2 Gy comparison, indicating that higher radiation doses elicit a broader spectrum of cell death and stress response pathways.

Next, we examined whether the pathways associated with exclusively differentially expressed proteins differed between the 2 Gy group and the 2 Gy + seco-DSA group. Specifically, we compared the 445 proteins unique to the 2 Gy group (Figure 5G) and the 392 proteins unique to the 2 Gy + seco-DSA group (Figure 5H). Both treatments showed a substantial number of proteins associated with autophagy, 15 in the 2 Gy group and 11 in the 2 Gy + seco-DSA group, and the same number of proteins related to the apoptotic process (8). However, the 2 Gy group had a notably higher number of cell cycle–related proteins (30) compared to the combination treatment (10). Interestingly, the 2 Gy + seco-DSA group exhibited a broader activation of cell death and stress-related pathways, suggesting a shift in the cellular response. Cell motility was associated with 8 proteins in 2 Gy + seco-DSA, double that of 2 Gy alone (4). Only the combination group showed proteins associated with senescence (2 proteins), whereas the 2 Gy group had none. Necrosis was linked to 4 proteins in the combination group, compared to just 1 in 2 Gy. The intrinsic apoptotic signaling pathway was associated with 4 exclusive proteins in the combination group, while only 2 were seen in 2 Gy. For DNA repair, the 2 Gy group had slightly more exclusive proteins (5 vs. 3 in 2 Gy + seco-DSA). While both treatments trigger similar core processes such as autophagy, the combination of 2 Gy + seco-DSA leads to a broader and more varied proteomic response, engaging additional pathways such as senescence, motility, necrosis, and intrinsic apoptosis.

At the 4 Gy dose, there was a higher number of differentially expressed proteins exclusive to the 4 Gy group (487) compared to the 4 Gy + seco-DSA group (152). This was also reflected in a greater number of differentially expressed proteins associated with the pathways (Figure 5I,J). The 4 Gy group had a higher number of uniquely expressed proteins associated with autophagy (13 vs. 3), apoptotic processes (11 vs. 4), and cell motility (7 vs. 3). Interestingly, 4 Gy + seco-DSA expressed fewer proteins involved in DNA repair (1 vs. 9) compared to 4 Gy alone. These findings suggest that 4 Gy radiation alone induces a broad proteomic response, engaging multiple pathways including cell cycle arrest, apoptosis, autophagy, and DNA repair, and that the addition of seco-DSA results in a smaller but potentially more focused set of differentially expressed proteins. This pattern suggests that seco-DSA may not redirect the cell toward entirely different death mechanisms but instead modulates the strength or regulatory balance of the existing stress response, potentially reinforcing checkpoint arrest or limiting repair and recovery mechanisms.

## 3. Discussion

GBM cells are resistant to both radiation and chemotherapy. GBM cell lines T98G and LN18 express high levels of O6-methylguanine-DNA methyltransferase, contributing to their marked resistance to FDA-approved alkylating agents such as temozolomide. To address this challenge, as discussed in our previous work, we first evaluated the potency of seco-DSA in these highly resistant GBM cells [18]. Our findings revealed that seco-DSA is highly potent as a standalone treatment against GBM cells; however, its non-selective cytotoxicity poses a significant limitation, as it also affects normal cells. To mitigate this limitation and improve therapeutic specificity, antibody-drug conjugates targeting tumor-specific antigens are being considered as a potential delivery strategy for seco-DSA. In addition, we investigated whether seco-DSA could function as a radiosensitizer to enhance the efficacy of radiation therapy in these radioresistant cell lines. The main goal was to determine whether a combined treatment approach, using low doses of seco-DSA and irradiation, could synergistically maximize tumor cell killing while minimizing systemic toxicity.

The first step was to determine and compare the radiation resistance of two GBM cell lines, T98G and LN18, under our experimental conditions. Colony formation assay data showed that the IC_50_ for T98G was 2.5 Gy, compared to 1.6 Gy for LN18, indicating that T98G is the more radioresistant of the two. This finding aligns with previously published work, which has also reported greater radiation resistance in T98G relative to LN18 [22]. The enhanced resistance of T98G may be attributed to its homozygous p53 mutation and mutant PTEN, whereas LN18 harbors a heterozygous p53 mutation and wild-type PTEN [23]. Additionally, both cell lines exhibited high α/β ratios, suggesting low sensitivity to fractionated doses and a limited capacity for sublethal damage repair. In T98G, the addition of seco-DSA resulted in additive radiation enhancement, whereas in LN18, it produced a synergistic effect when 0.004 nM and 0.008 nM seco-DSA were combined with 2 Gy of radiation. These findings suggest that seco-DSA enhances radiation response in both cell lines, but the nature of this enhancement differs based on their intrinsic genetic profiles and radiosensitivity. In T98G, the response was additive, consistent with its higher baseline resistance. In contrast, LN18 exhibited a synergistic response. This highlights the potential for genotype-specific radiosensitization strategies using seco-DSA in combination with radiation.

Similarly, both cell lines exhibited a dose-dependent decrease in cell proliferation. In T98G cells, combining the lowest concentration of seco-DSA (0.025 nM) with the lowest radiation dose (2 Gy) resulted in proliferation arrest comparable to that induced by the highest radiation dose alone (8 Gy). These results indicate that seco-DSA can significantly enhance the antiproliferative effects of radiation, especially at low concentrations, suggesting its potential to reduce the required radiation dose while maintaining therapeutic efficacy, particularly in resistant GBM cells like T98G. Additionally, combining the IC_50_ concentration of seco-DSA (0.28 nM) with 2 Gy and 4 Gy of radiation resulted in a synergistic decrease in cell proliferation, further supporting the antiproliferative effects of proton radiation through a synergistic mechanism when used in combination with seco-DSA at low proton radiation doses. In LN18 cells, combining the IC_50_ concentration of seco-DSA (0.075 nM) with 2 Gy of radiation reduced cell proliferation to a level comparable to that observed with 8 Gy of radiation alone. Moreover, all three concentrations of seco-DSA tested induced a synergistic antiproliferative effect when combined with the low radiation dose of 2 Gy, further highlighting the potential of seco-DSA to enhance radiosensitivity in GBM cells.

Radiation alone induced a dose-dependent decrease in the G1-phase population and a corresponding increase in G2/M-phase arrest in both cell lines. Treatment with seco-DSA alone also resulted in a substantial reduction in G1-phase cells, accompanied by G2/M-phase accumulation in both cell lines. When seco-DSA was combined with radiation, both cell lines exhibited a further reduction in the G1-phase population, significantly exceeding the effects of either treatment alone. Notably, G2/M-phase arrest was markedly enhanced under the combination treatment, particularly in LN18 cells, where the addition of any radiation dose to seco-DSA led to a significantly greater accumulation in the G2/M phase than was observed with monotherapy. Both radiation and seco-DSA independently induce G2/M-phase arrest, a common indicator of DNA damage response activation. However, their combined effect leads to a significantly greater accumulation of cells in the G2/M phase, particularly in LN18 cells, indicating a synergistic disruption of cell cycle progression. The pronounced depletion of the G1-phase population and enhanced G2/M arrest under combination treatment imply that seco-DSA may potentiate radiation-induced DNA damage or impair cell cycle checkpoints. This supports the potential utility of seco-DSA as an effective radiosensitizer, especially in tumors with intact G2/M checkpoint signaling.

Combined treatment with proton radiation and seco-DSA led to a radiation dose-dependent increase in both apoptosis and necrosis, with apoptosis being the predominant form of cell death. In T98G cells, statistically significant increases in apoptosis were observed at both 2 Gy and 8 Gy in the combination treatment compared to radiation alone. In LN18 cells, a significant increase in apoptosis was detected only at 8 Gy when comparing the combination treatment to seco-DSA alone, with no other significant differences observed in apoptosis or necrosis at lower doses. However, the levels of apoptosis and necrosis alone did not fully account for the extent of proliferation arrest observed in the combination treatments, particularly in LN18. This discrepancy suggests that reduced proliferation may primarily reflect reproductive cell death, as assessed by clonogenic survival, while additional cellular processes such as mitotic failure, permanent growth arrest, senescence, autophagy, or stress response pathways may contribute to the observed antiproliferative phenotype. In this context, the observed synergy is more consistent with checkpoint saturation and impaired cellular recovery following combined DNA damage, rather than enhanced activation of lethal cell death pathways. Together, these findings indicate that the dominant biological outcome of the combination treatment is non-apoptotic reproductive cell death, rather than acute apoptotic or necrotic elimination. Therefore, proteomic analysis was performed to gain a broader and deeper understanding of the molecular pathways modulated by the combined treatment in the LN18 cell line.

Within the proteomics data, several key pathways stand out as playing a major role in radiation damage. GO annotations for “cell motility”, “DNA repair”, “senescence”, “autophagy”, and “apoptotic process” pathways were modulated in the radiation + seco-DSA groups and correlate with the cell viability and synergy assays of this study. 2Gy + seco-DSA had more representation within the “apoptosis” and “apoptotic process” categories compared to radiation or seco-DSA alone but had less representation in the “DNA repair”, “DNA damage response”, and “cell cycle” categories compared to 2 Gy alone. This shift may reflect seco-DSA’s ability to modulate radiation-induced cellular stress, engaging a variety of cell death mechanisms in GBM cells. Whether this engagement inhibits or promotes each of the pathways discussed, has yet to be determined, as future studies regarding up- and downregulation of these proteins are needed.

The 4 Gy alone group had greater representation in every category when compared to the 4 Gy + seco-DSA and the seco-DSA alone groups. This suggests that while radiation alone induces dose-dependent diversity in proteomic changes, the presence of seco-DSA may amplify or broaden signaling at lower doses but streamline the cellular response at higher doses, potentially enhancing treatment specificity and reducing off-target stress signaling. The difference in the number of exclusive proteins between 4 Gy (487) and 4 Gy + seco-DSA (152) may not indicate a weaker effect but instead may reflect a more targeted response. Functionally critical proteins could be driving these cytotoxic effects, and seco-DSA might suppress certain radiation-induced pathways such as survival or repair mechanisms that are active in 4 Gy alone. Additionally, the overlap between 4 Gy and 4 Gy + seco-DSA is large (626 shared proteins), reinforcing that seco-DSA works within the radiation framework, not independently. Validation using additional biological replicates, statistical correction, and targeted molecular assays will be required to confirm the relevance of these candidate pathways and proteins.

A limitation of this study is the absence of a direct comparison between proton and photon irradiation. Consequently, while the present data define the effects of seco-DSA under proton irradiation conditions, they do not permit conclusions regarding proton specificity versus general radiosensitization. In addition, these findings are preclinical in nature and are intended to provide quantitative and pathway-level context for interpreting radiosensitization effects, rather than to imply clinical readiness. Normal tissue response, in vivo efficacy, and treatment tolerability were not evaluated in this study. Future studies incorporating direct comparisons between radiation modalities and deeper pathway-level interrogation may help further refine the mechanisms underlying the observed synergistic effects. Specific examples from this dataset include proteins such as HSP90 and its various isoforms. These proteins are important factors within the radiation biological effect [24]. HSP90 family proteins are notably upregulated in cancer and are linked to radiation and chemotherapy resistance [25]. Recent studies have suggested that attenuating HSP90 proteins could be an impactful target for enhancing the radiobiological effect [24]. Notably, in our study, TRAP1 and HSP90AB2, both HSP90 isoforms [26], are diminished exclusively, based on our threshold cutoff of LFC > 2, in our 2 Gy + seco-DSA protein group. Notably, at this radiation dose with seco-DSA treatment, the greatest synergy was observed. Only a few examples of potential synergistic proteins within our dataset are represented. Further studies are required to explore the role of these factors in the synergistic effect observed. This initial proteomics study provides the framework for further studies within these pathways. We plan to repeat proteomics experiments to find significantly differentially expressed proteins and select key factors to investigate and propose a mechanism of synergy with seco-DSA and radiation therapy.

## 4. Material and Methods

### 4.1. Cell Lines and Cell Culture

Human T98G (ATCC CRL—1690) and LN18 (ATCC CRL—2610) cell lines were cultured in EMEM cell culture medium (Genesee Scientific, El Cajon, CA, USA) containing 10% fetal bovine serum (FBS, Genesee Scientific, El Cajon, CA, USA) and DMEM/High Glucose cell culture medium (Cytiva, Marlborough, MA, USA) containing 5% FBS (Genesee Scientific, El Cajon, CA, USA), respectively. All cells were maintained at 37 °C and 5% CO_2_ [18].

### 4.2. Proton Radiation

Irradiations were performed at the James M. Slater, MD Proton Treatment & Research Center, Loma Linda University Health, Loma Linda, CA, USA. Cells were irradiated with 250-MeV protons at the center of the spread-out Bragg peak (SOBP) of the proton beam, with a dose rate of 1 Gy/min and doses of 2, 4, and 8 Gy. Irradiations were performed with a vertically incident proton beam entering the culture plates from below while plates were positioned horizontally, and culture medium was not changed following irradiation. Cell seeding density at the time of irradiation differed by assay and is specified in the corresponding experimental sections.

### 4.3. Seco-DSA Treatment

Stock solutions of seco-DSA (MedChemExpress, Monmouth Junction, NJ, USA) were prepared by dilution in dimethyl sulfoxide (DMSO) to a final concentration of 100,000 nM and stored at −80 °C. For each experiment, stock solutions were further diluted in cell culture media to achieve the required concentrations and added to the cells 8 h prior to irradiation. Control cells were treated with 0.5% DMSO [18].

### 4.4. Colony Formation Assay

Cells were seeded in triplicate in 6-well plates at densities of 800 cells per well for T98G and 1000 cells per well for LN18 24 h prior to seco-DSA treatment and incubated at 37 °C and 5% CO_2_. Eight hours following the seco-DSA treatment, cells were irradiated and subsequently fixed and stained after one to two weeks. Cells were fixed with ice-cold methanol and acetic acid (3:1) for 15 min and stained with 0.5% crystal violet for 2 h. Colonies were imaged with Coomassie blue detection on a ChemiDoc^®^ imaging system (Bio-Rad, Hercules, CA, USA) and analyzed with ImageJ software version 1.54g (National Institutes of Health, Bethesda, MD, USA) using the “ColonyArea” plugin [27]. Data were fitted to a single target/single-hit model (STM) using the equation SF = *e* (−D/D_0_). STM is employed to define the IC_50_ value of seco-DSA and DSA. In this equation, D is the delivered dose/concentration, and D_0_ is the dose/concentration that inhibits proliferation by 63% [18,28]. Data were also analyzed using the linear-quadratic model (LQM), with the following equation: SF = $e^{-(\alpha D+\beta D^{2})}$. LQM is a well-established model that uses the α, β, and α/β ratio to define cell line radiosensitivity. D is the delivered dose, α is a parameter representing lethal damage from a single-hit, and β is a parameter representing sublethal damage from multiple hits [29].

### 4.5. Cell Proliferation Assay

Cells were seeded in triplicate in 6-well plates at a density of 100,000 cells per well and incubated at 37 °C in a humidified atmosphere with 5% CO_2_. After 24 h, cells were treated with seco-Duocarmycin SA (Seco-DSA), and proton irradiation was administered 8 h post-treatment. Seventy-two hours following irradiation, cells were detached using 0.25% trypsin (Genesee Scientific, El Cajon, CA, USA), stained with 0.4% trypan blue solution, and counted using a Countess II automated cell counter (Thermo Fisher Scientific, Waltham, MA, USA). Cell viability was analyzed using two types of normalization. In the first analysis, proliferation was calculated relative to the untreated control group to assess the overall antiproliferative effects of radiation and seco-DSA. In the second analysis, proliferation values from combination treatments were normalized to radiation-only treatment at the same dose to specifically highlight the additional inhibitory effect of Seco-DSA when combined with radiation.

### 4.6. Cell Cycle Analysis

Cells were seeded in triplicate in 6-well plates at a density of 100,000 cells per well 24 h pre-seco-DSA treatment and incubated at 37 °C and 5% CO_2_. Cells were irradiated 8 h after seco-DSA treatment and then detached 72 h post-irradiation, washed with PBS, and fixed with 70% ice-cold ethanol for >1 h at −20 °C. Cells were stained using FxCycle^TM^ PI/RNase staining solution and quantified using flow cytometry with a MACSQuant^®^ Analyzer 10 cytometer (Miltenyi Biotec, San Diego, CA, USA). Data were analyzed using FlowJo software version 10.10.0 (Ashland, OR, USA). Intact cells were first gated based on forward and side scatter to exclude debris. Doublets were excluded using propidium iodide area versus width gating to isolate single-cell events. DNA content histograms were generated from singlet populations, and cell cycle phase distributions (G_1_, S, and G_2_/M) were quantified based on propidium iodide fluorescence intensity.

### 4.7. Apoptosis and Necrosis Assay

Cells were seeded in triplicate in 6-well plates at a density of 100,000 cells per well 24 h before adding seco-DSA and maintained at 37 °C in an atmosphere of 5% CO_2_. Following 8 h of seco-DSA treatment, the cells were irradiated and, 72 h later, they were detached using a 0.25% trypsin solution (Genesee Scientific, El Cajon, CA, USA). Triplicates of each treatment were prepared in a 96-well plate, and 150,000 cells were placed in each well. Cells were double stained using the Pacific Blue^TM^ Annexin V Apoptosis Detection Kit with 7-AAD (Biolegend, San Diego, CA, USA) and quantified using flow cytometry with a MACSQuant^®^ Analyzer 10 cytometer (Miltenyi Biotec, San Diego, CA, USA). Cells were analyzed using FlowJo^TM^ Software for Mac Version 10.10.0 (BD Life Sciences, Ashland, OR, USA). Intact cells were first gated based on forward and side scatter to exclude debris. Annexin V and 7-AAD signals were analyzed using density plots, and apoptotic and necrotic populations were identified based on quadrant analysis.

### 4.8. Proteomic Analysis Sample Preparation

Proteomic sample preparation and LC-MS/MS analysis were performed by the Proteomics Core, Institute for Integrative Genome Biology, University of California, Riverside, using established standard protocols. Cells were seeded in T-75 flasks and treated with seco-DSA, and the protein isolate was collected in a pellet. Samples were denatured with urea and reduced with dithiothreitol (DTT) at 37 °C, followed by an IAA alkylation. The buffer was exchanged for ABC, and trypsin was added for a >16 h incubation. A Speed-Vac was used for peptide concentration; then, they were desalted using C18 Zip Tips. Peptides were dried using a Speed-Vac and stored until LC-MS/MS analysis.

### 4.9. LC-MS/MS Data Acquisition

The fractions were resuspended in 20 μL of water with 0.1% formic acid. Peptide samples were separated by nano-LC and analyzed by online electrospray tandem mass spectrometry. The experiments were performed on an EASY-nLC 1200 system (Thermo Fisher Scientific, Waltham, MA, USA) connected to a quadrupole-Orbitrap mass spectrometer Orbitrap Fusion Tribrid Mass Spectrometry equipped with an EASY-Spray ion source. A 5 μL peptide sample was loaded onto the trap column (Thermo Fisher Scientific Acclaim PepMap C18, 75 μm × 2 cm) with a flow of 10 μL/min for 3 min and subsequently separated on the analytical column (Acclaim PepMap C18, 75 μm × 2 cm) with a linear gradient from 3% D to 37% D in 180 min. The column was re-equilibrated at initial conditions for 5 min. The flow rate was maintained at 300 nL/min, and the column temperature was maintained at 45 °C. An electrospray voltage of 2.2 kV versus the inlet of the mass spectrometer was used. The Orbitrap Fusion Mass Spectrometry was operated in the data-dependent mode to switch automatically between MS and MS/MS acquisition. Survey full scan MS spectra (*m*/*z* 375–1500) were acquired with a mass resolution of 60 K, followed by fifteen sequential high-energy collisional dissociations (HCDs). The AGC target was set to 400,000, and the maximum injection time was 100 ms. MS/MS acquisition was performed in an ion trap. The AGC target was set to 3000, and the isolation window was 1.6 m/z. Ions with charge states 2+, 3+, and 4+ were sequentially fragmented by HCD with a normalized collision energy (NCE) of 35%, and the fixed first mass was set at 100. In all cases, one microscan was recorded using dynamic exclusion of 30 s. The raw data were processed and analyzed using MaxQuant (version 2.1.4.0) with a homemade database.

### 4.10. LC-MS/MS Data Search

The raw data were processed and analyzed using MaxQuant (version 2.1.4.0) with a homemade database. Mass tolerances for precursor and fragment ions were 6 and 10 ppm; respectively, the minimum peptide length was 6 amino acids, and the maximum number of missed cleavages for trypsin was 2.

### 4.11. Protein Mass Spectrometer Analysis

Peak-intensity data were used for differential expression between control and 0.1 nM DSA-treated groups. Log-Fold Change (LFC) values were collected relative to control peak intensity. A baseline threshold of 2 LFC was chosen to qualify proteins for further analysis. Zero values were removed from the trial data and not included in the final analysis. The package clusterProfiler [30] and org.Hs.eg.db were used in R version 4.4.1 [31] to obtain biological process ontology annotations for differentially expressed proteins. Annotation reports were filtered for proteins involved in selected pathways. All proteins found differentially expressed and present in selected pathways were assigned arrows indicating a change in expression relative to control. Upset plots, Venn diagrams, and heatmaps were created using ComplexUpset, ggvenn, and pheatmap, respectively, for data visualization (R version 4.4.1) [32].

### 4.12. Statistical Analysis

All experiments, with the exception of the proteomics data, were performed in biological and technical triplicates. Proteomics analysis was performed as a single biological replicate (n = 1). The two-tailed unpaired *t*-test was used to determine statistically significant differences, and statistical significance was determined at a *p* value ≤ 0.05. The Bliss synergy score was calculated using SynergyFinder Plus (3.10.3) [19].

## Figures and Tables

**Table 1:** T98G α/β ratio and D_50_ value.

Linear-Quadratic Model	Alpha (α)	0.128 Gy^−1^
	Beta (β)	0.019 Gy^−2^
	α/β	6.9 Gy
Single-Target Model	D_50_	2.5 Gy

**Table 2:** LN18 α/β ratio and D_50_ value.

Linear-Quadratic Model	Alpha (α)	0.23 Gy^−1^
	Beta (β)	0.032 Gy^−2^
	α/β	7.2 Gy
Single-Target Model	D_50_	1.6 Gy

## Data Availability

The raw data supporting the conclusions of this article will be made available by the authors on request.
